# Foundations for Soft, Smart Matter by Active Mechanical Metamaterials

**DOI:** 10.1002/advs.202001384

**Published:** 2020-08-18

**Authors:** Maya Pishvar, Ryan L. Harne

**Affiliations:** ^1^ Department of Mechanical and Aerospace Engineering The Ohio State University Columbus OH 43210 USA

**Keywords:** active mechanical metamaterials, flexible electronics, smart materials, soft matter, soft robotics

## Abstract

Emerging interest to synthesize active, engineered matter suggests a future where smart material systems and structures operate autonomously around people, serving diverse roles in engineering, medical, and scientific applications. Similar to biological organisms, a realization of active, engineered matter necessitates functionality culminating from a combination of sensory and control mechanisms in a versatile material frame. Recently, metamaterial platforms with integrated sensing and control have been exploited, so that outstanding non‐natural material behaviors are empowered by synergistic microstructures and controlled by smart materials and systems. This emerging body of science around active mechanical metamaterials offers a first glimpse at future foundations for autonomous engineered systems referred to here as soft, smart matter. Using natural inspirations, synergy across disciplines, and exploiting multiple length scales as well as multiple physics, researchers are devising compelling exemplars of actively controlled metamaterials, inspiring concepts for autonomous engineered matter. While scientific breakthroughs multiply in these fields, future technical challenges remain to be overcome to fulfill the vision of soft, smart matter. This Review surveys the intrinsically multidisciplinary body of science targeted to realize soft, smart matter via innovations in active mechanical metamaterials and proposes ongoing research targets that may deliver the promise of autonomous, engineered matter to full fruition.

## Graphical abstract

An emerging field of active mechanical metamaterials establishes foundations for soft, smart engineered matter that may autonomously operate in diverse future applications. This Review surveys advances in mechanical design, material formulation, sensory mechanisms, and control integration that are strategically synthesized in active mechanical metamaterials. Ongoing and future research milestones are identified to characterize the path ahead toward autonomous, engineered matter.

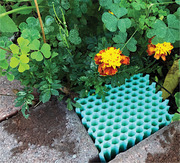

## 1 Introduction

In recent years, an accelerating scientific and technological interest has emerged to create functional, engineered material systems and structures that may emulate or exceed the versatile behaviors and properties of naturally occurring materials and organisms. Here, we term such functional engineered systems as soft, smart matter. While early embodiments of soft, smart matter demonstrated fundamental change of dynamic response via open‐loop control of fields, there is compelling vision in a future of fully autonomous, engineered matter that seamlessly operate in our natural and built environments. For example, one could fathom opportunities for autonomous, soft matter that traverse oceans to remove waste and report on water condition, that promote medical procedures and patient care by wound treatment and localized surgical support, or that promote urban growth and maintenance by adaptive and resilient infrastructural systems. To give rise to autonomous behavior, smart matters necessitates the incorporation of field‐responsive components, rational design principles, as well as sensing and control mechanisms governed by decision‐making networks.

Field‐responsive smart matter induces an internal flux in response to an applied field that tailors material and/or mechanical characteristics of the media. The applied field acts as a potential gradient on the material, where the potential may be voltage, pressure, temperature, and so on. The corresponding flux is then an electrical current, mechanical stress, heat flow, and so on. When responding to applied fields, a multitude of internal changes are possible in soft, smart matter. Materials that undergo changes to molecular arrangement, material phase, and crystal microstructure are termed active materials. Hydrogel, shape memory alloy, magnetostrictive, and piezoelectric materials are examples of active materials. By contrast, when fields do not yield bulk material transformations, we refer to these material systems as passive materials. Passive materials may also be field‐responsive, with notable examples being pressure‐driven, compliant solids seen throughout nature and in engineered systems. Together, active and passive field‐responsive materials are the enablers for a wide array of engineered smart matter.

Inspirations from nature have motivated myriad researchers to exploit the complex design principles of natural systems as a framework for the development of new types of autonomous, soft matter. These principles include synergistic interactions among multiple physics or hierarchical length scales; strategic use of periodicity, gradients, instabilities, and interfaces in design; unique relationships between wavelength and characteristic dimensions for non‐intuitive dynamic behavior; among others. In fact, such principles are central to the development in recent years of mechanical metamaterials: engineered matter that exhibits macroscopic behavior greater than the sum of bulk material parts. From hydrogel‐based metamaterials^[^ ^1^ ^]^ to liquid‐metal composites^[^ ^2^ ^]^ to electromagnetic lattices^[^ ^3^ ^]^ and beyond, there is now accelerated attention to combine field‐responsive materials with metamaterial principles inspired by natural examples to realize an emerging classes of active mechanical metamaterials. The recent examples of active mechanical metamaterials suggest high potential as a framework for soft, smart matter by virtue of new integrations among mechanical‐material properties, sensing, and control constituents.

This Review surveys the converging fields of science that are rapidly shedding light on innovative soft and smart matter enabled by active mechanical metamaterial principles. While this Review synthesizes key areas of development across an extraordinary range of disciplines, we recognize that not all specific research thrusts may be encapsulated here. As a result, we summarize three technical areas that have and continue to receive priority attention by scientists and scholars toward realizing soft and active mechanical metamaterials. These areas are (Section 2) metamaterial static and dynamic behaviors, (Section 3) functional materials and mechanisms, and (Section 4) the emerging field of active mechanical metamaterials. Following a summary of key developments in these respective technical areas, a discussion is given in Section 5 on the technical challenges that remain to surmount on the way to creating autonomous, soft matter.

## 2 Metamaterial Static and Dynamic Behaviors

Mechanical metamaterials exploit the design of microstructure, at length scales smaller than the whole material, to give rise to macroscale properties and behaviors that are not accessible by the bulk materials from which the metamaterial is derived. Many of these mechanisms are passive in that the meso‐ and microscale geometries culminate in unique behavior, rather than internal material or molecular reorganization, once acted on by external perturbations. The non‐natural characteristics of metamaterials include negative‐valued properties, unusual dynamic response, topological polarization, and non‐reciprocity, to name a few. Mechanical metamaterials have cultivated such capabilities through the use of microstructure possessing multiple length scales, graded and periodic design features, elastic instabilities, and unique interfaces. These mechanisms are ever present in natural materials and organisms. This section examines the state‐of‐the‐art investigations on mechanical metamaterials, exemplifying how mechanical designs of emerging metamaterials may serve as constituents in the formulation of future classes of autonomous, soft matter.

### 2.1 Manipulating Mechanical Properties

**Figure 1:**
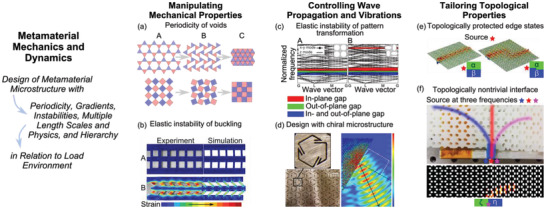
Mechanical metamaterials exploit microstructure morphology to give rise to exceptional properties and behaviors. a) Auxetic behavior cultivated in periodic arrangement of trihexagonal and square voids, showing deformation sequence from labels A, B, to C. b) Elastic buckling inducing reversible, repeatable shock energy dissipation mechanisms, evidenced by shear strain measured and determined by simulation before A and after B a uniaxial shock load. Adapted with permission.^[^ ^21^ ^]^ Copyright 2018, John Wiley & Sons, Inc. c) Metamaterial geometry transformations to tune band gaps. Adapted with permission.^[^ ^22^ ^]^ Copyright 2008, American Physical Society. d) Manipulation of wave refraction with chiral microstructure. Adapted with permission.^[^ ^23^ ^]^ Copyright 2014, Springer Nature. e) Tunable topologically protected edge states in a metamaterial by way of interfaces between *α*‐ and *β*‐lattices. Adapted with permission.^[^ ^24^ ^]^ Copyright 2018, American Physical Society. f) Wave localization and guiding of wave propagation in soft metamaterials by topologically nontrivial interface. Adapted with permission.^[^ ^25^ ^]^ Copyright 2018, Springer Nature.

When stretched uniaxially, conventional materials undergo a longitudinal extension along the stretch direction and lateral contraction perpendicular to the stretch direction. The negative of the ratio of such lateral to longitudinal strains is the material Poisson's ratio. In conventional bulk materials, the Poisson's ratio is a positive value between 0 and 0.5.^[^ ^4^ ^]^ Yet, materials exhibiting a negative Poisson's ratio, termed auxetic materials, have also been formulated by researchers. Significant interest to devise auxetic materials has grown in recent decades in part due to unique material strain upon loading and in part due to unique properties cultivated in auxetics. Greater indentation resistance and higher shear modulus are among the enhanced properties of auxetics, providing functional improvements in strength and durability.^[^ ^5^, ^6^ ^]^ Auxetic behavior has been identified and introduced in a wide range of mechanical metamaterials via periodic microstructures that possess auxetic unit cells geometries. **Figure** **1**a) schematically illustrates two examples of structures, made of periodic arrangement of trihexagonal^[^^]^ and square voids.^[^ ^8^ ^]^ These exemplary auxetics transition between porous shapes, Figure 1a) label A, to a deformed solid geometry, Figure 1a) label C. A wide variety of auxetic structures are explored for mechanical metamaterials including re‐entrant units,^[^ ^9^, ^10^ ^]^ chiral structures,^[^ ^11^, ^12^, ^13^ ^]^ bow‐tie elements,^[^ ^14^ ^]^ star shaped perforations,^[^ ^15^, ^16^ ^]^ circular void patterns,^[^ ^17^ ^]^ perforations with ancient geometric motifs,^[^ ^18^ ^]^ sinusoidally architected beams,^[^ ^19^ ^]^ and origami inspired patterns such as Miura‐ori.^[^ ^20^ ^]^

Elipe and Lantada^[^ ^26^ ^]^ cataloged auxetic behavior in 2D and 3D metamaterial unit cell geometries, revealing that the smallest Poisson's ratio around −1.8 is associated with re‐entrant cuboid microstructures. Using chiral unit cells or by pyramidal or square grid microstructure patterns, the Poisson's ratio may range from −0.1 to −0.9, resulting in a maximum volume reduction of 25–40%.^[^ ^26^ ^]^ In addition to tailoring microstructure, it was recently found that the value of Poisson's ratio is also dependent on the extent of material deformation.^[^ ^12^ ^]^ For instance, at large strains the microstructure deforms in a mechanically nonlinear regime, resulting in strain‐dependent auxetic behavior.^[^ ^12^ ^]^ To maximize auxetic response, Chen and Zheng^[^ ^27^ ^]^ proposed multi‐material metamaterials with spatially varying stiffness to induce a Poisson's ratio ranging from ‐7 to 0 when subjected to tensile strain up to 20%. Two main deformation mechanisms that lead to auxetic behavior are: i) rotation of unit cell microstructure upon uniaxial or biaxial metamaterial deformation and ii) unit cells with re‐entrant shape.^[^ ^28^, ^29^ ^]^ Thus, by an insightful selection of parameters in the design of mechanical metamaterials that cultivate such distinct deformation mechanisms, the Poisson's ratio and relative shape change may be tailored.

Elastic instabilities, and the associated negative stiffness property, are likewise often integral in the formulation of mechanical metamaterials. Unlike auxetic behavior that is rare in nature, elastic instabilities are key mechanisms for skeletal muscle force generation,^[^ ^30^ ^]^ for rapid motion in the plant kingdom,^[^ ^31^ ^]^ and for high‐rate force generation in the animal kingdom, as observed in earwigs,^[^ ^32^ ^]^ mantis shrimp,^[^ ^33^ ^]^ and hummingbirds,^[^ ^34^ ^]^ to name a few. Negative stiffness involves opposing directions of displacement and force, often a result of energy storage and release.^[^ ^35^ ^]^ As such, negative stiffness does not exist permanently unless constrained or stabilized with positive stiffness, such as by embedded negative stiffness inclusions in elastic matrices or within truss networks.^[^ ^36^, ^37^ ^]^ For mechanical metamaterials, the elastic instability of snap‐through buckling is widely leveraged^[^ ^38^, ^39^ ^]^ to provide non‐natural macroscale material behavior including shape reconfiguration,^[^ ^18^, ^40^ ^]^ surface control,^[^ ^41^ ^]^ and extreme static and dynamic behaviors.^[^ ^21^, ^42^, ^43^, ^44^ ^]^ For instance, in an archetypal elastomeric metamaterial composed of uniform vertical beams interfaced with a horizontal beam, Figure 1b), elastic beam buckling is harnessed to facilitate large shock energy dissipation.^[^ ^21^ ^]^ Additionally, Rafsanjani et al.^[^ ^42^ ^]^ exploited periodic unit cells in an elastic mechanical metamaterial with reversible snap buckling for tunable tensile behavior, including large change to surface topography from wavy‐ to diamond‐like patterns. Similar unit cells were considered in sandwich composites as means for large energy absorption due to plastic deformation of the buckling microstructure.^[^ ^45^ ^]^ Hewage et al.^[^ ^46^ ^]^ identified a “double‐negative” mechanical metamaterial that utilized interlocking elastic unit cells that resulted in auxetic shape change and negative stiffness at the macroscale, demonstrating a modular approach to realizing such non‐natural material behavior. Origami‐inspired metamaterials are likewise invested with tunable mechanical properties and dynamic behavior controlled by the manifestation and extent of negative stiffness introduced by implementing Miura‐ori, waterbomb, and twist folding patterns.^[^ ^47^, ^48^, ^49^, ^50^ ^]^ Typically, this class of mechanical metamaterials are realized by first constructing the planar tessellation patterns in the unfolded states, so that folding operations into 3D forms leads to the negative stiffness property.^[^ ^51^, ^52^, ^53^ ^]^ In this way, structural compressive modulus was reversibly reprogrammed by exploiting bistability in Miura‐ori origami pattern structures.^[^ ^54^ ^]^

Elastic instability is often exploited to transform the microstructure geometry in mechanical metamaterials. Uniaxial or biaxial compression and tension acting on cellular solids may trigger dramatic pattern transformations beyond critical loads, resulting in transition between chiral to achiral configurations or among multiple collapsed states.^[^ ^55^, ^56^, ^57^ ^]^ By such transformations, Poisson's ratio,^[^ ^7^ ^]^ mechanical properties,^[^ ^58^, ^59^ ^]^ and wave propagation^[^ ^60^ ^]^ characteristics of mechanical metamaterials may be tailored. Motivated by these observations, Coulais et al.^[^ ^61^ ^]^ articulated how nonlinearities deformations of metabeams, beam‐like structures with metamaterial microstructures, resulted in discontinuous buckling, where the postbuckling stiffness increases from negative values when the aspect ratio of elliptical microstructures increases. El‐Helou and Harne^[^ ^58^ ^]^ explored functional gradients in microstructure to tailor mechanical properties by inducing multiple elastic instabilities in a mechanical metamaterial. The authors uncovered fundamental relationships between the intricate elastomeric beam network of the metamaterial and linear elastic spring networks, helping create a straightforward design tool for mechanical properties and critical points of instability.^[^ ^58^ ^]^ Such methodology to achieve pattern transformations on the basis of elastic instability is often realized in 2D cellular metamaterials with square,^[^ ^7^ ^]^ triangular,^[^ ^62^ ^]^ trihexagonal and rhombitrihexagonal^[^ ^7^ ^]^ arrays of cellular voids. Yet, the principle extends to patterned lattices more generally so long as antagonistic networks are established. For instance, 2D beam lattices,^[^ ^63^ ^]^ 3D porous Bucklicrystal networks,^[^ ^64^ ^]^ 3D Buckliballs,^[^ ^65^ ^]^ and patterned cylindrical^[^ ^66^, ^67^ ^]^ shell structures exhibit shape transformations through elastic instabilities intrinsic to the cellular solid networks. In general, these studies demonstrate that auxetic behavior and collapse phenomena may be cultivated by exploiting multistable internal microstructures that capitalize on small perturbations to result in large shape conformation or reactive force.

Researchers have also uncovered how mechanical metamaterials may exhibit negative compressibility transitions, which is the non‐intuitive coincidence of longitudinal deformation in response to an applied longitudinal force.^[^ ^68^, ^69^ ^]^ Nicolaou and Motter^[^ ^68^ ^]^ proposed metamaterial architecture, based on nonlinear interaction potentials between constituent materials, which allows for this non‐natural behavior. Qu et al.^[^ ^70^, ^71^ ^]^ created poroelastic metamaterials that isotropically expand in the presence of increasing external pressure. By exploiting microstructures with intricate connectivity and elements that store and release energy, such tunable properties including negative compressibility enable mechanical metamaterials to react and change shape in a vast range of ways in response to environmental stimuli.

When compressed, bulk materials bulge. On the other hand, generations of mechanical metamaterials have been devised that exhibit lateral twisting and rotation when subjected to compression. Frenzel et al.^[^ ^72^ ^]^ enabled twist‐coupled deformation in a 3D metamaterial by leveraging rotationally symmetric and chiral unit cells. Chirality was also utilized in the cylindrical metamaterial devised by Wu et al.^[^ ^73^ ^]^ to lead to linear changes of metamaterial rotation angle in response to increasing axial compression. Waterbomb origami‐based mechanical metamaterials have also been investigated for unique twisting behavior under uniaxial compression, revealing how the twist originates from the central unit cells and successively spreads row to row toward the ends.^[^ ^74^ ^]^ Such twist‐coupled behaviors offer opportunities for engineered matter with versatile actuated shape change, such as for grippers and robotic manipulators.

The microstructures of mechanical metamaterials facilitate a wealth of mechanical properties that induce versatile shape change and load bearing response desirable for future embodiments of soft, smart matter. Such behaviors may serve as key ingredients to synthesize controllable combinations of static and dynamic response in autonomous engineered materials.

### 2.2 Controlling Elastic Wave Propagation and Mechanical Vibrations

One may find innumerable examples of natural materials possessing exceptional dynamic behaviors in consequence to unique microstructures. For example, spider webs exhibit unusual strength and vibration damping by virtue of the periodic radial construction with a nonlinear viscoelastic silk filament,^[^ ^75^ ^]^ while the ordered and hierarchical branching of trees is strategically scaled to best attenuate aerodynamic‐induced vibrations.^[^ ^76^ ^]^ Likewise, cultivating enhanced dynamic behaviors in mechanical metamaterials is a consistent research target, providing multiple opportunities for dynamically resilient soft matter platforms.

One dynamic property sought by researchers is the formation of band gaps in the dispersion behavior of mechanical metamaterials. Band gaps are frequency ranges where elastic waves may not propagate through the material. The Bragg scattering phenomenon has been employed to induce band gaps through wave scattering at material interfaces. By this phenomenon, the wavelength that is attenuated has similar length scale as the characteristic structural dimension, which limits band gap formation by Bragg scattering to higher frequency ranges for most metamaterial applications.^[^ ^77^, ^78^, ^79^ ^]^

On the other hand, low frequency waves and vibrations are often more damaging to engineered structures and materials due to the resulting larger displacements and strains. As a result, sub‐wavelength attenuation mechanisms, such as resonant unit cells, are widely investigated.^[^ ^38^ ^]^ In this regard, metamaterials composed from lattice or truss networks have drawn much attention.^[^ ^80^ ^]^ The existence and characteristics of a locally resonant band gap are governed by the lattice topology.^[^ ^81^, ^82^ ^]^

Resonant mechanisms may include unit cells containing locally resonant inclusions, inducing a frequency dependent and negative effective mass behavior corresponding to band gap existence.^[^ ^77^, ^83^ ^]^ Qureshi et al.^[^ ^84^ ^]^ demonstrated that significant wave attenuation is achieved in the band gap resulting from cantilever‐in‐mass metamaterials, with wave attenuation capability determined by the unit cell dimensions and material properties. Jiao and Gonella^[^ ^85^ ^]^ investigated the wave propagation in a nonlinear spring‐mass chain coupled with a locally resonant cubic nonlinear foundation, discovering intriguing band gap switching mechanisms corresponding to nonlinear bifurcation. For finite‐sized metamaterials, vibrations result from the presence of stress or strain perturbations. In this case, the band gap principle established for wave propagation through infinite materials also leads to vibration suppression in finite mechanical metamaterials.^[^ ^86^, ^87^, ^88^, ^89^, ^90^, ^91^ ^]^ Sugino et al.^[^ ^92^ ^]^ identified the relationship between the number of such locally resonant inclusions and the resulting vibration‐based band gap behavior, helping bridge understanding between the infinite media band gap and finite metamaterial vibration attenuation.

Periodic, cellular elastomeric materials are another widely investigated foundation for wave blocking metamaterials. The transformation among metamaterial cross‐sectional topology due to elastic instability and collapse is often leveraged to govern band gap formation and tuning.^[^ ^22^, ^60^, ^62^ ^]^ The use of deformation to tune and transform the band gap of periodic structures with a square array of voids was shown by Bertoldi and Boyce,^[^ ^22^ ^]^ Figure 1c. Zhang et al.^[^ ^93^ ^]^ reported that by adjusting the layout of the periodic stacked Miura‐origami unit cells in origami metastructure, the band gap can be effectively tailored. The activation of wrinkling patterns in multi‐material interfaces was considered by Rudykh and Boyce^[^ ^94^ ^]^ to be another approach to control band gaps by reversible elastic instability. Because the wrinkling in soft matter cultivates collective wavy patterning to the material, researchers exploited this principle to study other soft matter metamaterials that induce wavy deformation fields upon compressive loading, likewise creating means to control band gap characteristics.^[^ ^95^ ^]^ Reversible elastic buckling has also been leveraged to amplify broadband dissipative effects in mechanical metamaterials subjected to vibration and impact. Cui and Harne^[^ ^44^ ^]^ confirmed the relationship between critical constraints near buckling and the largest growth of broadband vibration suppression in elastomeric metamaterials. Researchers have also scrutinized mechanisms of high rate impact mitigation in elastomeric and polymeric mechanical metamaterials including buckling internal elements,^[^ ^21^, ^96^, ^97^ ^]^ quantifying the dramatic reduction of transmitted force or acceleration due to temporary storage of strain energy in the elastic lattice microstructure.

In addition to governing the ability for waves to propagate, there are a number of exciting opportunities for mechanical metamaterials that use strategic microstructure to control the direction of wave propagation, such as for sensing, energy harvesting, and communications.^[^ ^23^, ^80^, ^98^ ^]^ Tol et al.^[^ ^99^ ^]^ enabled an omnidirectional wave focusing mechanism through a metamaterial lens with Luneburg type geometry. Zhu et al.^[^ ^23^ ^]^ manipulated sub‐wavelength wave refraction in an elastic metamaterial with chiral microstructure, Figure 1d. Dong et al.^[^ ^100^ ^]^ realized negative refraction of both longitudinal and transverse waves in an anisotropic metamaterial with architecture identified through topology optimization. Anisotropy is also central in the metamaterials formulated by Celli and Gonella^[^ ^101^ ^]^ that guide distinct wave modes on the basis of relaxed unit cell symmetries in design. In the limits of finite wave amplitudes on metamaterial lattices, the directional nature of wave propagation in mass‐spring hexagonal lattices is correlated with applied strain amplitude.^[^ ^102^ ^]^

A number of mechanisms that result in non‐natural mechanical behavior in metamaterials likewise lead to exceptional dynamic response. Band gaps, vibration attenuation, shock mitigation, and directional wave propagation culminate by strategically designing and implementing mechanical metamaterials. As a result, a strong foundation is laid for both mechanically and dynamically resilient engineered media to be employed in the formulation of active controlled soft matter.

### 2.3 Tailoring Topological Properties

Recently, new frontiers are being forged in generations of mechanical metamaterials that exhibit wave fields confined to propagate along boundaries, edges, and interfaces. This behavior is fundamentally distinct from Rayleigh or Stonely‐Scholte wave propagation predicted by classical elastodynamic theory,^[^ ^103^, ^104^ ^]^ thus suggesting opportunities for novel concepts for wave‐protected structures and highly absorptive surfaces, to name a few potential applications. The inspiration for these mechanical metamaterials is found in quantum mechanics and condensed matter physics.^[^ ^105^ ^]^ Starting from the efforts of Haldane^[^ ^106^ ^]^ who predicted the possibility of edge states in electronic materials, topologically protected edge states became a significant focus of research in quantum mechanics. Original works associated these properties with breaking of time‐reversal symmetry,^[^ ^107^ ^]^ although Kane and Mele^[^ ^108^ ^]^ later identified topological modes in graphene that were not bound by such requirement. In this way, analogues to topological phases in other physics were proposed, and later identified for elastic lattices^[^ ^109^ ^]^ and periodic mechanical metamaterials.^[^ ^110^ ^]^

A variety of remarkable behaviors are cultivated in topological metamaterials, including localized floppy edge modes,^[^ ^111^ ^]^ non‐reciprocity and topologically protected wave propagation,^[^ ^110^ ^]^ transformation between deformable to rigid configurations,^[^ ^112^ ^]^ and selective buckling and reconfiguration.^[^ ^113^, ^114^ ^]^

Many studies have given attention to Maxwell lattices for which the average number of constraints equals the degrees of freedom in the system. Thus, Maxwell lattices are on the border of mechanical instability.^[^ ^109^ ^]^ Maxwell lattices may include square and Kagome architectures in 2D, and cubic and pyrochlore lattices in 3D.^[^ ^115^ ^]^ Floppy modes may occur in these lattices, associated with zero energy modes that permit kinematic edge rotation.^[^ ^111^ ^]^ Rocklin et al.^[^ ^111^ ^]^ proposed a design approach whereby twisting of relative periodic structures in a Kagome lattice transitioned the topological polarization of the metamaterial by moving the locations at which the floppy edge modes occur. Paulose et al.^[^ ^116^ ^]^ applied topological defects on the interior of a deformed Kagome lattice to create anisotropic localization of waves. Origami‐ and kirigami‐inspired metamaterials have been shown to likewise exhibit such wave localization by exploiting different folding or cut patterns at interfaces, establishing a unique vision for deployable topological mechanical metamaterials.^[^ ^117^ ^]^

Asymmetric or non‐reciprocal wave transport and topologically protected wave propagation without backscattering are often considered in the formulation of topological mechanical metamaterials.^[^ ^25^, ^110^ ^]^ Ma et al.^[^ ^118^ ^]^ articulated the role of edge modes on asymmetric wave transport in topological lattices, showing how excitation at the floppy edge prohibits wave penetration into the lattice. Goldsberry et al.^[^ ^119^ ^]^ took a different approach to break reciprocity. The authors exploit mechanical modulation of pre‐strain in a honeycomb structure to generate non‐reciprocal wave phenomena in metamaterials with time‐ and space‐dependent effective material properties.

The manipulation of wave propagation is possible by edge‐to‐edge joining of two elastic systems with distinct topological invariants (also called Chern numbers), which results in the generation of topologically protected edge states at the interface.^[^ ^25^, ^110^, ^120^ ^]^ For instance, the existence of such topological interface states was demonstrated by Liu and Semperlotti^[^ ^24^ ^]^ in material systems assembled from distinctly deformed states of a lattices, labeled as *α* and *β* lattices in Figure 1e. By changing the interface geometry between the *α* and *β* lattices, a symmetric or anti‐symmetric edge state is formed at the domain wall guiding waves along the interface. Figure 1f. In a similar way, Li et al.^[^ ^25^ ^]^ presented tunable topological states in soft elastic metamaterials by combining two periodic honeycomb patterns. The authors^[^ ^25^ ^]^ found that depending on the frequency of elastic waves exciting the interface in relation to the flat band frequency, the elastic wave may be localized on the interface or may propagate through each domains, thus providing a frequency dependence to topological protection, Figure 1f.

Recently, Chen et al.^[^ ^121^ ^]^ uncovered three elastic topological phases that may be realized in a mass‐spring honeycomb lattice, where each non‐trivial phase corresponds to distinct symmetry breaking induced by mass‐spring parameter selections. Guo et al.^[^ ^122^ ^]^ realized topologically protected edge states and propagation of elastic waves around acute edges in a 2D metamaterial based on a unit cell of the 2D pentamode structure with the honeycomb lattice arrangement. Topological protection is moreover robust to parametric perturbation, as demonstrated through design variations in a Kagome lattice exhibiting topologically protected edge states.^[^ ^123^ ^]^

The foundation of soft, smart matter is a versatile and passive mechanical‐material frame that interacts with the environment. Through hierarchical, synergistic, and periodic microstructures, researchers have established a wealth of techniques by which to invest mechanical metamaterials with exceptional mechanical and dynamic properties that distinguish such engineered matter from bulk material counterparts. Given such opportunities to realize non‐natural characteristics in mechanical metamaterials, the opportunity to control the properties in real‐time through external fields or integrated mechanisms is a natural pursuit toward the direction of autonomous, soft, smart matter.

## 3 Functional Materials and Mechanisms

Many living organisms use sensory feedback to identify the state of the system and to identify actions necessary to return to an equilibrium once acted on by external perturbations. Likewise, the robust and autonomous operation of engineered systems requires the ability to sense the system behavior and to control the subsequent response. Over the years, researchers have made extraordinary progress to devise classes of functional materials and devices that leverage multiphysics coupling to detect and react against applied mechanical perturbations. This section summarizes the advancements made to synthesize functional materials and mechanisms that enable mechanical shape change, actuation, and sensing capability, with emphasis on those that may contribute to future soft, smart matter.

### 3.1 Field‐Responsive Materials and Devices

Field‐responsive materials undergo intrinsic phase, molecular, or chemical changes in response to applied fields including electric field, magnetic field, thermal gradient, light, pH, and ionic strength.^[^ ^124^, ^125^, ^126^, ^127^ ^]^ This class of responsive media has attracted considerable interest in the formulation of smart and functional matter. Researchers have pursued numerous avenues whereby compliant materials and structures are composed from field‐responsive media to realize functionalities such as sensing and actuation. Oftentimes, because macroscopic behavior is an overarching target, both the micro‐ as well as mesoscale system formulation are simultaneously taken into consideration with smart material integration. Here, we summarize how field‐responsive materials are recently harnessed to achieve novel functionalities in compliant smart matter that may serve as a basis for future active metamaterials.

**Figure 2:**
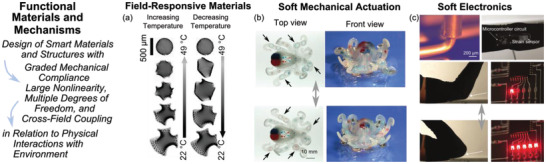
Functional materials and mechanisms composed from field‐responsive media leverage graded mechanical compliance, nonlinearity, multiple degrees of freedom, and cross‐filed coupling for versatility in function. a) Patterned gel sheets in the aqueous medium, undergoing 3D shape transformation in response to temperature change. Adapted with permission.^[^ ^133^ ^]^ Copyright 2012, American Association for the Advancement of Science. b) Octobot‐like robot, powered by combustion and controlled by microfluidic logic to alternate between two states. Adapted with permission.^[^ ^148^ ^]^ Copyright 2016, Springer Nature. c) Flexible electronic materials created from 3D printed conductive ink that is used to monitor joint bending. Adapted with permission.^[^ ^149^ ^]^ Copyright 2017, John Wiley & Sons, Inc.

Field‐responsive hydrogels represent a fascinating class of functional polymers owing to an ability to absorb and retain high water content.^[^ ^1^, ^128^, ^129^ ^]^ For example, the poly(*N*‐isopropylacrylamide) (PNIPAm) is a thermosensitive material showing a substantial volume change when heated above a lower critical solution temperature as water is expelled during a phase transition.^[^ ^130^ ^]^ Accordingly, water‐sorption‐induced swelling can be utilized for actuation by applying an inhomogeneous field such as a gradient of temperature, or by using inhomogeneous materials exploiting a gradient of swelling properties.^[^ ^131^ ^]^ For instance, Ma et al.^[^ ^132^ ^]^ used a water gradient to obtain asymmetric deformation in a composite polypyrole‐based film, driving film locomotion. Alternatively, Kim et al.^[^ ^133^ ^]^ demonstrated 3D shape transformation due to temperature change in patterned gel sheets composed of highly cross‐linked dots embedded in a lightly cross‐linked matrix of a swellable polymer, shown in **Figure** **2**a.

Shape memory polymers are active materials that exhibit programmable shape recovery in response to environmental stimuli such as heat or light. The shape memory effect is a thermomechanical functionalization of the material termed programming that may capitalize on phase transitions, thermoplasticity, or other mechanisms of material transformation.^[^ ^134^, ^135^ ^]^ In a temperature‐responsive shape memory polymer, the cycle of programming is initiated by applying external stress at a temperature above the thermal transition point of material causing a temporary deformation of the material. The unstable state is fixed by cooling to a temperature below the thermal transition point, followed by release of the external stress. The recovery to the original shape is then achieved by heating to a temperature above thermal transition point of material. This approach is termed one‐way actuation. In addition, Behl et al.^[^ ^136^ ^]^ demonstrated reversible and reprogrammable actuation via crystallization/melting in a ribbon of thermally controlled shape‐memory polymer with a concertina shape. Jin et al.^[^ ^137^ ^]^ uncovered unique shape‐morphing behaviors from a polymer sheet with photo‐defined reversible actuation and thermally triggered permanent shape reconfiguration mechanisms.

Piezoelectric materials are capable of generating electrical field from mechanical stress, and vice versa.^[^ ^138^, ^139^ ^]^ Due to the polarization of ions in a crystal that has no center of symmetry, a piezopotential is formed in the crystal by applying a mechanical stress. An example use of the piezoelectric mechanism was demonstrated in ref. ^[^ ^140^ ^]^ where flexible PZT (lead zirconate titanate) harvesters were designed to capture electrical power from motions of the heart, diaphragm, and lung. Furthermore, the elastic properties of the a human epidermal layer of skin were determined using stretchable networks of mechanical actuators and sensors constructed from PZT nanoribbons.^[^ ^141^ ^]^ Piezoelectric polymers such as polyvinylidene fluoride (PVDF) and its copolymer, poly(vinylidene fluoride–trifluoroethylene) (PVDF–TrFE), are also of interest due to enhanced compliance compared to lead‐based piezoelectric materials.^[^ ^142^ ^]^ For instance, Lee et al.^[^ ^143^ ^]^ presented a nanogenerator able to covert low‐frequency bending of the human arm into electrical current using a hybridization approach of two piezoelectric materials, zinc oxide nanowires, and PVDF polymer. Yet, in general piezoelectric actuators are limited due to their small deflections compared to shape memory polymer actuators although piezoelectric materials mechanically response to electric stimulus at much higher rates of change than shape memory or hydrogel materials.^[^ ^144^ ^]^

Numerous other active materials are under close investigation, for instance ionic polymer‐metal composites,^[^ ^145^ ^]^ magnetostrictive materials,^[^ ^146^ ^]^ and moisture responsive materials.^[^ ^147^ ^]^ In fact, by discovering new material combinations and fabrication techniques, a variety of sensors and actuators may be developed for complex mechanical operations.

Compliant mechanisms are milli or meso length scale devices with actuated motion and sensing facilitated by strategic flexibility of smart material components. Compliant mechanisms are here classified as passive structures yet are field‐responsive by selective use of smart materials for sake of sensing and actuation. These platforms represent steps in the direction toward small‐scale, smart material‐based active structures that have inspired mechanical metamaterials. Compliant mechanisms inspire novel solutions for applications including medical devices, robotics, microelectromechanical systems, and deployable structures.^[^ ^150^, ^151^, ^152^, ^153^, ^154^, ^155^ ^]^

Baker and Howell^[^ ^156^ ^]^ designed a compliant bistable micromechanism controlled by thermal actuation. Moulton and Ananthasuresh^[^ ^157^ ^]^ explored an electro‐thermal‐compliant actuation (ETC) approach for MEMS devices, where the mechanism underlying shape control is differential thermal expansion tuned by the MEMS shape. Alternatively, an integrated thermal actuator may be realized by multi‐material layers or structure sections where mismatch between thermal expansion coefficients results in shape change upon device heating and cooling.^[^ ^158^, ^159^ ^]^ Integrated sensing approaches along with correlations between compliant mechanism dynamic behavior are central to the ability to control a compliant mechanism in real‐time using sensory feedback.^[^ ^160^ ^]^ Messenger et al.^[^ ^161^ ^]^ integrated piezoresistive sensing into a microdisplacement transducer for closed‐loop feedback control of thermal MEMS actuators. Magnetoelastic coupling was exploited by Butler et al.^[^ ^162^ ^]^ for an origami‐based mechanism with multi‐input mechanical advantage. Alternatively, bifurcations in compliant mechanism behavior, such as due to elastic instabilities, have been harnessed as means to detect both continuous gradients of response as well as thresholds of extreme events, including by electrostatic and piezoelectric coupling.^[^ ^163^, ^164^ ^]^

Autonomous soft, smart matter requires means to convert controlled stimuli into change of shape, mechanical properties, and reactive force. Advances in field‐responsive materials and devices have established a multitude of techniques whereby active and passive materials may provide sensing and actuation functionality amenable to metamaterial formulations. One specific technical focus has risen up so prominently in this spirit through broad initiatives to promote advanced manufacturing and automation: soft mechanical actuators. This technical field is surveyed in detail in the following section.

### 3.2 Soft Mechanical Actuation

Engineered autonomous matter has significant foundation in the fields of robotics, manufacturing, and automation. Consequently, the recent surge of investigations on soft robots has built up broad inspirations and methodologies to devise controllable structures involving continuous deformation of a soft frame.^[^ ^165^, ^166^, ^167^, ^168^ ^]^ These compliant actuators have the potential to be resilient in applications with conventional access limitations, and may be safer for human interaction. On the other hand, the soft body dynamics exhibits nonlinearity, elasticity, and many degrees of freedom, posing new challenges compared to rigid robots. As a result, a considerable body of the literature surrounding the science of soft robots has developed in recent years, creating numerous inspirations for the broader class of soft, smart matter. The following paragraphs highlight the key advancements and knowledge derived from the emerging field of soft robotics.

Feinberg et al.^[^ ^169^ ^]^ demonstrated an early realization of a soft robot formed via a bilayer material system with cultures of cardiac muscle tissue sheets on flexible PDMS thin film substrates. Depending on the tissue architecture, thin film shape, and the applied electrical field stimulation, the soft robotic actuators could perform a wide range of functional behaviors such as gripping, walking, and swimming with fine spatial and temporal control. Control of soft polymeric aquabots was achieved with electric and magnetic fields by Kwon et al.,^[^ ^170^ ^]^ who exemplified the use of pressurized microfluidic chamber volume change that governed movement, sensing/signaling, and capture/transport/release functionalities. Using the principle of muscular hydrostat units, which are structures that conserve volume during contractions, an octopus‐like soft robot arm was formulated and controlled by shape memory alloy wire networks embedded within the soft elastomeric matrix.^[^ ^171^ ^]^ A unique approach based on coupling among monopropellant fuels and microfluidic logic is devised in ref. ^[^ ^148^ ^]^ for a pneumatic octobot‐like robot, Figure 2b, able to autonomously alternate between two actuation states without controller intervention.

Soft robots may be conceived as compliant structural‐material frame that houses conventional rigid driving elements such as the controller, power source, and actuators. This approach necessitates careful synthesis of design at the interfaces between rigid and soft elements, unless interface failure occurs. To overcome this limitation, a combustion‐powered jumping robot was created with orders of magnitude gradient of elastic modulus to mismatch of compliance at interfaces.^[^ ^172^ ^]^ Palagi et al.^[^ ^173^ ^]^ proposed the use of photoactive liquid‐crystal elastomers to power and control shape changes in soft robots when acted on by structured monochromatic light. Actuation by vacuum pressure is also investigated in a class of origami‐inspired soft robots^[^ ^174^ ^]^ that alternate among twisting‐contraction and twisting‐bending actions based on the vacuum application, leading to locomotion and gripping robot functionalities.

Due to the distributed compliance and complex nature of continuous deformation observed for soft robotics, researchers are also establishing data‐driven models that inform soft robot controllers to maintain reference states and perform target functions.^[^ ^175^ ^]^ Nakajima et al.^[^ ^176^ ^]^ used a machine‐learning approach, called reservoir computing, to create closed‐loop control for robust maintenance of a soft robot arm motions. For robust, self‐sensing soft robots, an inductance‐based system was presented in ref. ^[^ ^177^ ^]^ and demonstrated improved joint deformation monitoring of a bellows‐driven soft robot than those with traditional inertial measurement units.

As one step toward a broader formulation of soft, smart matter, the innovations in emerging soft mechanical actuation provide inspirations for techniques to leverage fluidic, mechanical, magnetic, and pneumatic approaches to control soft structural‐material shape and create mechanical force. Once coupled with versatile range of mechanical and dynamic behavior and via automated control networks, the principles of soft robotics create a compelling vision of autonomous engineered matter.

### 3.3 Flexible Electronic Materials and Systems

The development of soft, engineered matter may often require electrical signal transmission whether used for sensing and detection or for transmitting actuator commands. The use of electrical signaling is especially desirable given synthesis of soft and smart matter with digital electronics and networking solutions. Consequently, electrical conductors that stretch according to large, continuous conformations of a soft matter are required. Unfortunately, most electrical conductors are rigid metals ill‐suited to need. As a result, a recent surge of research is devoted to establish flexible electronic materials and material systems that sustain electrical conductivity during high‐strain deformations such as stretching, folding, or bending.^[^ ^178^, ^179^ ^]^ These materials are useful for electrical applications in different fields such as soft robotics, conformable skin sensors, and wearable or implantable devices. There are two approaches explored to date to realize flexible electronics: i) exploiting large mechanical deformation of conventional rigid conductors, and ii) utilizing dispersion and deposition of conductive fillers into stretchable elastic matrices. This section summarizes the distinct approaches to realize flexible electronic materials and significant discoveries in this important area of research that will contribute to future soft, smart matter.

Mechanics‐driven methods of creating flexible electronics exploit large elastic deformations of conductive materials in ways that mitigate yield or fatigue failure for the same strains that would otherwise destroy rigid conductors. For example, Lacour et al.^[^ ^180^ ^]^ used wavy strips of gold film on an elastomeric PDMS membrane and found that the strips remain conducting even when stretched by as much as 22% strain. Wavy silicon ribbons bonded to a PDMS substrate were also found to be reversibly stretched and compressed to a greater level of strain, near 30%, without visible damage.^[^ ^181^ ^]^ Herringbone‐shaped silicon nanomembranes on elastomeric substrates were shown to permit large biaxial strain, thus extending the mechanics‐driven concept to multi‐dimensional flexibility.^[^ ^182^ ^]^ These advancements inspired Fan et al.^[^ ^183^ ^]^ to explore fractal‐based layouts of silicon nanomembranes on soft substrates, using patterning such as Peano, Greek cross, and Vicsek. Rogers et al.^[^ ^184^ ^]^ provides a Review of the numerous techniques by which stretchable electronics may be realized by integrating micro‐/nanostructured inorganic and organic electronic materials with elastomeric substrates. A key advantage of the mechanics‐driven method to devise flexible electronics is that the conductors retain nearly the peak conductivity of the bulk materials from which the conformal variants derive. This characteristic is in striking contrast to the flexible electronic materials and systems resulting from the second approach explored to date.

Rather than using mechanical deformation of micro‐/nanoscale conductors, the second approach to realize stretchable electronic materials employs conductive fillers dispersed within an elastic matrix or substrate. In this way, percolation is required to yield a conductive material:^[^ ^185^ ^]^ that is, sufficient contact of filler particles must be achieved to create continuous and low‐resistance electrical connections through the media. The conductive fillers explored to date range from carbon nanotubes, to silver and gold, to non‐toxic liquid metals, and more, using particle sizes ranging from nanoscale structures to millimeter size liquid metal channels.

For instance, Sekitani et al.^[^ ^186^ ^]^ used single‐walled carbon nanotubes (SWNTs) dispersed through a copolymer matrix to realize a stretchable composite film with uniaxial stretchability of 134% while maintaining electrical conductivity. Nanotube thin films deposited on acrylic adhesive substrates are explored in ref. ^[^ ^187^ ^]^ and reported to remain conductive for strains up to 700%. A stretchable composite composed of silver (Ag) and PDMS was proposed by Larmagnac et al.,^[^ ^188^ ^]^ which was stretched at strains exceeding 100% while maintaining conductivity using 23–25 vol% Ag concentration. Polyurethane‐gold stretchable nanocomposite conductors, capable of undergoing a large strain of 486%, are investigated in ref. ^[^ ^189^ ^]^. Polyurethane also serves as the matrix in which Ag microflakes are dispersed for a 3D‐printable flexible electronic ink, Figure 2c, explored by Valentine et al.^[^ ^149^ ^]^ Figure 2c illustrates that a wearable and stretchable electronic device monitors large strain associated with joint bending.^[^ ^149^ ^]^ Sears et al.^[^ ^190^ ^]^ harnessed Ag‐polyurethane mixtures as inks to be placed on mechanical metamaterials, giving a first vision of flexible electronic metamaterials where conductivity through the engineered material system is governed by mechanical and dynamic strains at both local and macroscopic length scales.^[^ ^190^ ^]^

Non‐toxic liquid metals, such as gallium‐indium (GaIn) alloys, are also used as dispersed conductive fillers or as direct electrical conduits. Liquid metals are distinct with respect to other classes of conductive fillers in that liquids do not support shear stress although they conserve volume (under moderate compression). As a result, liquid metals, often GaIn based, are widely considered as a foundation to realize soft and stretchable electronic materials.^[^ ^191^ ^]^ These benefits have instigated the development of highly stretchable conductors, consisting of patterned, millimeter‐scale eutectic GaIn droplets embedded in a 3D nanostructured PDMS matrix.^[^ ^192^ ^]^ Fassler and Majidi^[^ ^193^ ^]^ introduced an alternative approach whereby percolation is realized by a stochastic micro‐scale GaIn and elastomer mixture (liquid metal elastomer: LME) that achieves conductivity once the host elastomeric matrix tears due to mechanical sintering. Markvicka et al.^[^ ^194^ ^]^ explored how damage to a conducting LME may be mitigated due to the intrinsic nature for the percolative liquid metal micro‐network to recoalesce around damage sites, such as punctures, tears, and so on. Thrasher et al.^[^ ^195^ ^]^ capitalized on acrylate ligands bound to liquid metal microparticles that are subsequently polymerized to create conducting liquid metal networks demonstrating excellent electrical power delivery and minimized concern over oxidation. Oxidation is also alleviated in the liquid metal nanoparticle graft to hydrophobic polymer proposed by Lin et al.,^[^ ^196^ ^]^ revealing particular promise for biomedical flexible electronics applications due to the stability of the material in aqueous environments.

The neural channels that permeate many living organisms permit centralized command and processing, while actuation is communicated to local, distributed motion‐ and force‐generating mechanisms. To emulate such practice of global‐local control in monolithic architectures, flexible conductive materials are required for soft, smart matter. A recent growth of interest in flexible conductive materials gives scientists numerous concepts to realize synergistic interactions among material, mechanical, and electrical physics for new generations of flexible electronics.

Research in the broad areas of functional materials and mechanisms has established understanding to guide the intelligent use of programmable compliance and integrated control, sensing and electrical power delivery conductors, and microstructure composition with ordered macroscale shape change. These advancements deliver vast foundation upon which to build up innovative active metamaterials with integrated sensory‐control mechanisms.

## 4 Active Mechanical Metamaterials

As described in introductory remarks, an active mechanical metamaterial realizes active functionality by exploiting field‐responsive materials whether those materials are active (bulk material composition change when subjected to fields) or passive (no change to bulk material). In this regard, a range of stimuli have been explored to govern mechanical metamaterial properties, such as electric fields, chemical or thermal gradients, pressure differences, and more. By virtue of synthesizing intelligent microstructured mechanical design, control authority, and potential sensory mechanisms, active mechanical metamaterials are emerging precursors to autonomous soft, smart matter. The following section summarizes the acceleration of research targeted to realize active mechanical metamaterials, identifying how the principles from metamaterial mechanics and from functional materials and mechanisms are finding union for the first time, on the road to autonomous materials.

**Figure 3:**
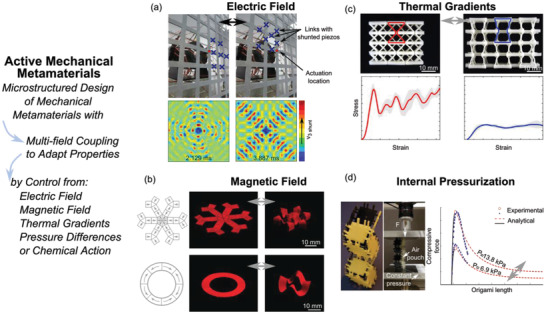
Active mechanical metamaterials exploit multi‐field coupling by integrating material, mechanical design, sensing, and control strategies to actively adapt shapes and properties. a) Electric field control of a cellular lattice to tailor band gap properties seen in the dispersion relations. Adapted with permission.^[^ ^201^ ^]^ Copyright 2018, American Physical Society. b) Magnetic field control of soft structures with programmed ferromagnetic domains to obtain complex 3D shape. Adapted with permission.^[^ ^204^ ^]^ Copyright 2018, Springer Nature. c) Thermal gradient based control of cellular structures made of shape memory polymers to switch topologies and mechanical properties. Adapted with permission.^[^ ^205^ ^]^ Copyright 2019, Elsevier. d) Pressure controlled in a fluidic origami cellular structure to actuate folded states. Adapted with permission.^[^ ^206^ ^]^ Copyright 2016, American Physical Society.

Piezoelectric materials have been adopted in recent metamaterials to convert energy of electric field into mechanical action, such as to control bending impedance and tailor wave propagation.^[^ ^197^ ^]^ For instance, Zhu et al.^[^ ^198^ ^]^ implemented shunted piezoelectric patches in an acrylic tubular metamaterial to tune band gap behavior at a subwavelength scale. An active metamaterial plate with periodic cantilever‐mass microstructures interfaced with shunted piezoelectric patches has also exemplified band gap control of both longitudinal and bending waves.^[^ ^199^ ^]^ Bergamini et al.^[^ ^200^ ^]^ devised a periodic hybrid metamaterial consisting of elastic beam and collocated piezoelectric elements that, when shunted, provided significant wave attenuation at frequencies tuned according to the shunt circuit parameters. Spatial wave control capability is demonstrated by Celli et al.^[^ ^201^ ^]^ in a lattice‐resonator network using piezoelectrics and resistor‐inductor shunts, **Figure** **3**a. The authors discovered means to override intrinsic anisotropy of wave guiding in the lattice via the selective activation of the electromechanical resonators.^[^ ^201^ ^]^ Piezoelectric material implementations of active mechanical metamaterials benefit from the intrinsic self‐sensing property of piezoelectric structural integrations,^[^ ^202^ ^]^ although the brittle nature of piezoelectrics and their high bulk modulus^[^ ^203^ ^]^ pose some challenges in the synthesis of soft matter.

Magnetically responsive materials are also explored as means to govern properties and shape of active mechanical metamaterials with the application of magnetic field. Harne et al.^[^ ^207^ ^]^ demonstrated large magnetic field tuning of mechanical and dynamic behavior in cellular metamaterials having iron particles stochastically dispersed in the elastomer and aligned during the elastomer curing process. Furthermore, manufactured structures are recently shown to exhibit intricate shape changes and programmable auxetic response by the strategic ferromagnetic domain alignment that leads to ordered deformation when subjected to uniform magnetic fields,^[^ ^204^ ^]^ Figure 3b.

Researchers have also shown techniques to exploit combinations of bending and folding in magnetically‐actuated, square metamaterial unit cells that exhibited as much as 200% stiffness change under application of magnetic field.^[^ ^208^ ^]^ Tuning of internal resonances is studied in ref.^[^ ^209^ ^]^ to manipulate wave propagation in a metamaterial frame consisting of a lattice structure and a pair of embedded electromagnets for each unit cell. Magnetic field control of magnetoelastic metamaterials offers desirable non‐contact means for governing properties and mechanical shape, thus potentially obviating the need for flexible conductor networks, yet comes with a trade‐off of highly nonlinear response due to dipole‐dipole interaction.^[^ ^210^ ^]^

Thermal control of soft matter metamaterials also has broad potential applications due to the miniaturization opportunities afforded to thermal sensors and actuators. Silverberg et al.^[^ ^49^ ^]^ fabricated origami structures based on a square‐twist pattern and showed that self‐folding can be actuated by heating or cooling of temperature‐responsive polymer‐gel. Wagner et al.^[^ ^205^ ^]^ introduced programmable active lattice cellular structures made of shape memory polymers that can switch between a stretch‐dominated topology, Figure 3c left, to a bending‐dominated topology, Figure 3c right, under a heat stimulus. By switching between topologies, the mechanical properties of lattice structures are tuned in real time. Ware et al.^[^ ^211^ ^]^ introduced novel voxelated liquid crystal elastomers (LCE) that transition between pre‐defined metamaterial shapes based on the thermal environment and topological defects created in LCE polarization. In addition, the multistable characteristics in multimaterial square‐twist origami structures were exploit by Wang et al.^[^ ^212^ ^]^ to trigger thermodriven self‐deployment. Similarly, Tang et al.^[^ ^213^ ^]^ demonstrated adaptive programmable kiri‐kirigami metamaterials able to govern structural angles by thermal stimulus.

Researchers have also found promise to control metamaterial response through hydraulic or pneumatic pressurization of enclosed chambers in the metamaterial frame. Unlike previous structures, this class of adaptive structures are made of passive material systems but can be activated/programed upon pressurization in a purely passive fashion. Lazarus and Reis^[^ ^66^ ^]^ studied soft, auxetic cylinders patterned with an array of voids that reversibly collapsed when pressurized or evacuated with hydraulic fluid, resulting in tunable flexure or twist motion. Li et al.^[^ ^206^ ^]^ created fluidic origami that morphs shape and tunes stiffness by exploiting the correlation between folding motion and the enclosed internal fluid volume, controlled by fluid pressurization and collapse of origami structure, Figure 3d. Motivated by these observations, the actuation capabilities of a plant‐inspired fluidic origami cellular structure activated by internal pneumatic pressure were demonstrated by Sane et al.^[^ ^214^ ^]^ and optimal fluidic origami designs for actuation were identified. Origami‐inspired artificial muscles, assembled from zig‐zag origami structure, flexible elastomeric skin, and a fluid medium, demonstrated multidimensional shape change under negative pressure controls.^[^ ^215^ ^]^ In addition, Overvelde et al.^[^ ^216^ ^]^ investigated an origami mechanical metamaterial with enclosed pneumatic chambers that permitted control of metamaterial shape, volume, and stiffness. Pressurization of chambers is well suited as a control mechanism for soft matter based metamaterials, as has multiple analogues in nature such as nastic plant motion,^[^ ^217^ ^]^ motivating close consideration of means to optimize such motion and force control strategies in ongoing research.

As research begins in earnest to devise soft, smart matter, the early formulations of active mechanical metamaterials probe the new challenges intrinsic in the multi‐disciplinary system synthesis. Overall, these first efforts exemplify the promise of integration of material, mechanical design, sensing, and control for new generations of active mechanical metamaterials.

## 5 Technical Challenges and Future Research Prospects

**Figure 4:**
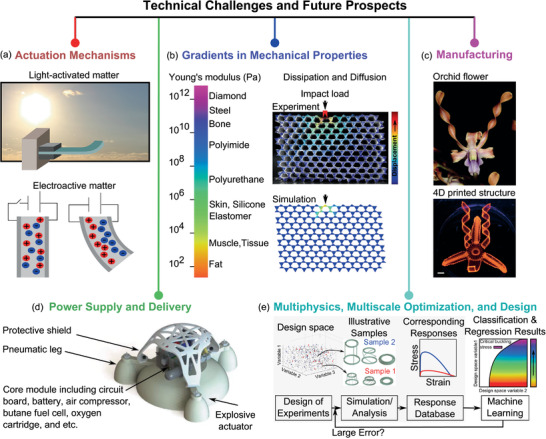
Technical challenges in the formulation of autonomous engineered matter. a) Harnessing diverse actuation mechanisms to control shape and properties. b) Exploitation of material modulus gradients and/or dynamic dissipation and diffusion mechanisms for compliant structures. c) development of facile manufacturing techniques for production of complex structures, e.g., using four‐dimensional (4D) printing to create a structure with orchid flower morphologies. Adapted with permission.^[^ ^218^ ^]^ Copyright 2016, Springer Nature. Scale bar, 5 mm. d) Providing integrated power supplies and energy delivery. Adapted with permission.^[^ ^172^ ^]^ Copyright 2015, American Association for the Advancement of Science. e) Building up multiphysics, multiscale optimization and design, such as a Bayesian machine learning approach. Adapted with permission.^[^ ^219^ ^]^ Copyright 2019, John Wiley & Sons, Inc.

By leveraging hierarchy, multiple physics, patterning, interfaces, and other intelligent design concepts, soft and smart matter established on foundations of active mechanical metamaterials may harness a suite of naturally‐inspired capabilities that suggest great potential once synthesized into self‐contained active material embodiments. Key challenges that remain to formulate autonomous engineered matter are depicted in **Figure** **4** and are further described in the following section, along with possible approaches to surmount the technical hurdles ahead.

### 5.1 Actuation Mechanisms

Many living organisms, especially animals, use chemical energy conversion to lead to force generation and shape change, such as ATP hydrolysis in skeletal muscle.^[^ ^220^ ^]^ Hydraulic mechanisms of actuation are also abundant in the plant kingdom, especially in tandem with the storage and release of elastic energy.^[^ ^221^, ^222^ ^]^ Distributed actuation is oftentimes employed by living organisms to create force and motion. Controlling the behavior of future soft, smart matter may be assisted by leveraging these principles. Mechanical metamaterials consisting of cellular void patterns possess an intrinsic compatibility for distributed hydraulic or pneumatic actuation through a practice of pressurizing or evacuating the void passages.^[^ ^223^ ^]^ Such a vision for distributed pneumatic actuation has also been applied to a bellows‐driven compliant robot.^[^ ^177^ ^]^ Numerous avenues remain to be explored for distributed actuation methods in active mechanical metamaterials, although the classes of actuators that permit distributed actuation is limited to date. For instance, light‐activated matter suggests uniquely sustainable actuation opportunities by exploiting solar radiation,^[^ ^224^ ^]^ Figure 4a top. Similarly, development of electroactive materials that leverage electrical stimulus to modulate behavior are readily synthesized with modern controllers, offering ease of system integration, Figure 4a bottom. Chemical actuation of metamaterials is explored at micro‐/nanoscales,^[^ ^225^ ^]^ but has yet to be brought to macroscale functionality of interest for future soft, smart matter applications suggesting great opportunities remain to further establish chemically driven operation of autonomous mechanical metamaterials. To date, more conventional engineering‐focused actuation approaches have been considered for engineered soft matter based on active mechanical metamaterials, such as piezoelectrics, hydrogels, magnetoactive materials, shape memory materials, and more. In these approaches, the energy density may be high to exert large authority over motion and force, but an inherent modulus mismatch results when integrated into soft matter.

### 5.2 Gradients in Material and Mechanical Properties

The mismatch of modulus is a more pervasive challenge to formulate soft, smart matter than merely for selection of actuation mechanisms, Figure 4b. Unlike central nervous systems and control networks in biological matter, self‐contained active mechanical metamaterials may require digital logic to process sensed signals and system response and to determine control actions. Digital devices are often fabricated with silicon which has a bulk modulus many orders of magnitude greater than most soft materials considered in a vision of soft, smart matter. Consequently, gradients from stiff to soft modulus are required to alleviate separation at interfaces.^[^ ^226^ ^]^ Indeed, this concern is independent of autonomous mechanical metamaterial context and has been a long‐standing challenge in biomedical applications.^[^ ^227^ ^]^ The acceleration of flexible electronics research^[^ ^184^, ^191^ ^]^ is a forecast of the projected need to incorporate effectively rigid components in compliant material frames. Excepting a few innovative approaches for entirely soft material logic that are likewise deserving significant research continuation,^[^ ^148^ ^]^ scientists and engineers alike have great motivations to devise transformative approaches^[^ ^228^, ^229^ ^]^ to overcome the modulus mismatch between digital logic devices and soft matter.

Damping and diffusion are inherent in the dynamic behavior of materials. Considering smart matter implementation may involve elastic waves, shock, signal transmission, and other dynamic responses, a detailed understanding of how waves and vibrations dissipate and transmit through mechanical metamaterials is essential to acquire in order to design for robust functionality. Topologically protected edge states and non‐reciprocal wave propagation are attractive properties to cultivate in active mechanical metamaterials, such as for switchable signal transmission and stealth characteristics. Yet, the presence of wave dissipation and diffusion challenges utilization of these phenomena. For example, Figure 4b shows the impact response of a soft topological metamaterial that may be diminished by way of energy dissipation and diffusion mechanisms. As a result, researchers have begun exploring injected control action as a means to accommodate and offset wave energy loss to recover such properties as non‐reciprocity.^[^ ^230^, ^231^ ^]^ Such loss compensation has been generalized theoretically for mechanical metamaterials by Torrent et al.^[^ ^232^ ^]^ although experimental realizations remain to be devised for the numerous potential implementations of soft, smart matter. Overcoming wave diffusion may be achieved by virtue of exploiting topological edge states^[^ ^24^ ^]^ so long as damping is minimized or counteracted. Innovative approaches to release energy stored in elastic instabilities have also been considered to amplify wave generation in actuation modes.^[^ ^233^ ^]^ Damping and diffusion will continue to demand attention in new formulations of soft, smart matter particularly when wave propagation behavior is of importance.

### 5.3 Manufacturing

Current active mechanical metamaterials have relied heavily on additive manufacturing (AM) and 3D printing methods to realize laboratory demonstrations of field‐responsive metamaterials.^[^ ^234^ ^]^ To create 3D micro/nano lattice‐based metamaterials, one widely used approach is 3D extrusion printing where the bulk material is heated and deposited through pressurized nozzles.^[^ ^235^ ^]^ Extrusion printing permits multi‐material layering and customization of viscoelastic properties.^[^ ^236^ ^]^ Alternatively, inkjet 3D printing deposits polymer droplets onto a flat or powder bed. In the deposition‐based printing methods, the quality of the printed metamaterial greatly depends on the printing process, printing environment, and polymer ink properties. Other common AM techniques leveraged to date for metamaterial fabrication include photopolymerization of resin with UV light or lasers and powder‐bed fusion that exploits sintering or melting of polymer powder via a laser source.^[^ ^53^ ^]^ Likewise for these methods, printing protocols and material properties strongly govern success and quality of the resulting metamaterial. The concept of 4D printing advances AM techniques by harnessing temporal change of a 3D printed structure due to environmental stimulus. In this regard, hydrogel inks containing stiff anisotropic cellulose fibrils were designed by Gladman et al.^[^ ^218^ ^]^ so that a water immersion of the 3D printed architecture induced swelling and shape change. Figure 4c shows a 4D printed structure inspired by orchid flower morphologies, exhibiting four configurations: bending, twisting, and ruffling corolla surrounding the central funnel‐like domain.^[^ ^218^ ^]^ By combining the automation‐ready approaches of pick‐and‐place with AM methods,^[^ ^149^ ^]^ sensing, actuation, and digital controller components may be embedded accurately and repeatably inside active mechanical metamaterials. While the scientific development of soft, smart matter remains in a relatively early stage, it is prudent for potential end‐use cases to be on the minds of researchers. For instance, medical applications may require customized field‐responsive metamaterials in low volumes fulfilled by AM methods,^[^ ^237^ ^]^ whereas soft, smart matter intended for capturing particulates to clean a water supply may necessitate millions of units, thus encouraging mass production methods of manufacture. This forward‐thinking approach has already served to promote commercialization and societal impact of optical and electromagnetic metamaterials^[^ ^238^ ^]^ and may be favorably adopted by scientists engaged in the formulation of concepts for active mechanical metamaterials.

### 5.4 Power Supply and Delivery

The chemomechanical and fluidic pressure modes of energy conversion in living organisms are not currently the primary modes of inducing motion and force generation in active mechanical metamaterials. Instead, electromechanical, electrochemical, piezoelectric, electrodynamic, hydraulic, and other techniques are widely utilized to control engineered materials and systems. As an example, a photograph of a soft robot with pneumatic control components and microcontroller circuit from ref. ^[^ ^172^ ^]^ is presented in Figure 4d, powered using combustion. Achieving sufficient power density for electrically governed smart matter is a classic challenge due to balance of energy consumption and storage with state‐of‐the‐art batteries and efficient power electronics.^[^ ^239^ ^]^ The power consumption for microprocessors, the energy required for processing and storing information, in addition to the minimum amount of mechanical energy required by the system to function are the limiting factors for energy efficiency and speed of movement. For self‐sufficient autonomous smart matter, battery supplies are therefore likely required to control actuators, pumps, and other electronic components,^[^ ^240^ ^]^ although recharging or energy harvesting methods are necessary to sustain functionality.^[^ ^241^ ^]^ Additional research attention to chemical^[^ ^148^ ^]^ or optical/solar^[^ ^242^ ^]^ modes of power conversion is highly deserving given potential to free soft, smart matter from electrical grid reliance for truly sustainable and autonomous operation.

### 5.5 Multiphysics, Multiscale Optimization, and Design

From a design perspective, the convergence of mechanics, material, sensing, and control principles in soft, smart matter has posed an intricate problem, especially in the optimization domain. The nonlinear and non‐affine interactions amongst the myriad design variables must therefore be understood to inform optimization processes. To date, a number of researchers have tackled the design process of multiphysics and multiscale mechanical metamaterials. For example, topological optimization of a functionally graded cellular composites is investigated in ref. ^[^ ^243^ ^]^ accounting for auxetic behavior and mechanical stiffness characteristics. Level‐set methods and asymptotic homogenization topology optimization are employed in ref. ^[^ ^244^ ^]^ to obtain optimal periodic micro‐architectured materials with target elasticity tensor and Poisson's ratio. Multi‐objective topology optimization has also been studied to balance effective bulk modulus with thermal conductivity properties for periodic composites.^[^ ^245^ ^]^ These investigations are important steps toward establishing formal design processes of mechanical metamaterials. On the other hand, autonomous soft, smart matter will possess a substantially greater number of design variables and functional objectives. To this end, machine learning techniques, such as by Bayesian methods,^[^ ^219^ ^]^ may provide the necessary breadth of data assessment to reveal feasible and optimal methods of active mechanical metamaterial formulation, Figure 4e. To shed light on the underlying physics of such engineered smart matter, machine learning approaches such as sparse identification of nonlinear dynamical systems^[^ ^246^ ^]^ may be exploited, thus helping extend beyond identification of advanced next‐generation materials^[^ ^247^ ^]^ and serving as foundation to uncover principles of design and implementation of active mechanical metamaterials.

## 6 Conclusions

Soft, smart matter represents a significant step forward toward engineered systems that help promote, augment, and elevate quality of life and supportive technologies. Through this Review, we have discussed and summarized how the constituents intrinsic to active mechanical metamaterials realize promising technical foundation for future autonomous, soft matter. Beyond the technical challenges identified here, an unsaid requirement to fulfill such a grand vision is the inherent need for multidisciplinary scientific teams to come together to devise innovations that bridge the fields of chemistry, materials science, mechanical and electrical engineering, manufacturing, and more. Genuinely compelling solutions to the myriad fundamental problems identified through this Review may be proposed and explored by diverse teams of scientists who culminate in synergistic ideas and technical capability. Given the versatility of multidisciplinary research teams to innovate holistic solutions to large scientific challenges, the future of soft, smart matter founded on active mechanical metamaterials may be close on the horizon.

## Conflict of Interest

The authors declare no conflict of interest.
